# A Case of Kikuchi's Disease Without Cervical Lymphadenopathy

**DOI:** 10.1155/2022/2943233

**Published:** 2022-12-05

**Authors:** Shinya Tomori, Seigo Korematsu, Taichi Momose, Yasuko Urushihara, Shuji Momose, Koichi Moriwaki

**Affiliations:** ^1^Department of Pediatrics, Saitama Medical Center Saitama Medical University, Kamoda, Kawagoe Saitama 350-8550, Japan; ^2^Department of Pathology, Saitama Medical Center, Saitama Medical University, Kamoda, Kawagoe Saitama 350-8550, Japan

## Abstract

**Background:**

Kikuchi's disease with only extracervical lymphadenopathy is rare. *Case Presentation*. A 15-year-old male has presented with a fever lasting more than 1 week and right axillary lymphadenopathy. An axillary lymph node biopsy revealed coagulation necrosis, nuclear decay products, infiltration of histiocytes, and enlarged lymphocytes; he was diagnosed with Kikuchi's disease. The only four adult patients with Kikuchi's disease presenting without cervical lesions have been previously reported.

**Conclusion:**

This is the only pediatric case of Kikuchi's disease presenting without cervical lymphadenopathy. Kikuchi's disease should be included in the differential diagnosis even in cases of extracervical lymphadenopathy alone.

## 1. Introduction

Kikuchi's disease, also called Kikuchi-Fujimoto disease or histiocytic necrotizing lymphadenitis, is a benign condition of unknown cause characterized by a fever and cervical lymphadenopathy [1]. It was first reported from Japan by Kikuchi and Fujimoto in [2, 3]. The disease is thought to involve a viral immune response, and many triggers have been proposed, including Epstein-Barr virus, human herpesvirus 6, human herpesvirus 8, human immunodeficiency virus, parvovirus B19, paramyxovirus, parainfluenza virus, Yersinia enterocolitica, and toxoplasma [4]. Fever combined with cervical lymphadenopathy is the most typical clinical feature of Kikuchi's disease [5]. Other sites of lymphadenopathy other than the cervical lymph nodes include the axilla, mediastinum, groin, abdomen, and pelvis [5]. Kikuchi's disease without cervical lymphadenopathy is rare [6, 7].

We herein report a case of Kikuchi's disease presenting only with axillary lymphadenopathy, along with a review of previous reports.

## 2. Case Presentation

A 15-year-old boy born to healthy Japanese parents developed a fever, headache, and malaise 13 days before admission. He visited his local doctor one week prior to admission due to his persistent fever, and a blood test was performed but revealed no abnormalities. He was prescribed acetaminophen, but as the fever did not resolve, he visited our hospital three days prior to admission and was prescribed ampicillin/clavulanic acid. However, he was admitted due to his continued fever. He had never been scratched by a cat or other animals.

On admission, his consciousness was clear. His temperature was 39.0°C, and he had no respiratory disturbance or tachycardia. There was a 3-cm smooth, mobile, and tender lymphadenopathy in the right axilla. There were no scratches or injuries on his right arm. Other physical examination findings were normal. Laboratory results are shown in Table 1. Contrast-enhanced computed tomography (CT) showed multiple lymphadenopathies in the right axilla but no solid tumor.

**Figure 1:**
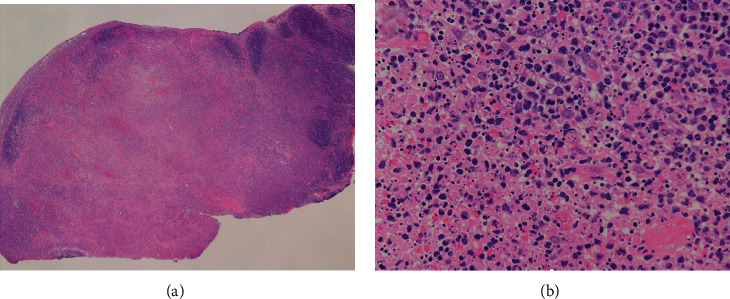
A histopathological image of axillary lymph node biopsy performed on the fourth day of admission. (a) Low power field, the central part of the lymph node was necrotic. (b) Hyper power field, macrophages with numerous nuclear karyorrhectic debris were observed in the necrotic tissue. The biopsied lymph node showed no neutrophilic infiltration.

Initially, we suspected pyogenic lymphadenitis and started treatment with cefepime and vancomycin. However, since the fever did not resolve and there was a discrepancy between inflammatory findings on blood tests and clinical findings, an axillary lymph node biopsy was performed on the fourth day of admission in order to differentiate malignant lymphoma. The pathology showed indistinct lymph node structure and an infiltrate of large lymphocytes and histiocytes from cortical to paracortical areas. In addition, the central part of the lymph node was necrotic, and macrophages with numerous nuclear karyorrhectic debris were observed in the necrotic tissue. The biopsied lymph node showed no neutrophilic infiltration (Figure 1(a) and 1(b)). Based on these pathological findings, a diagnosis of Kikuchi's disease was made.

Antimicrobial therapy was discontinued on day 9 of hospitalization. On the same day, small erythematous plaques without fusion appeared on the extremities and trunk. The patient was diagnosed with a skin rash associated with Kikuchi's disease. Olopatadine was administered because itching also appeared. On the 13th day of hospitalization, the patient's symptoms improved, and the inflammatory findings became negative, so he was discharged. Since then, the patient's symptoms have not flared up.

## 3. Discussion

Differential diagnoses of regional lymphadenopathy include hematologic malignancies, cancer metastases, hypersensitivity syndromes, infections, connective tissue disorders, atypical lymphoproliferative disorders, granulomatous disease, and Kikuchi disease. Among these, axillary lymphadenopathy is generally differentiated from infections (staphylococcal and streptococcal skin infections, cat scratch disease), sarcoidosis, and malignancies (breast cancer, lymphomas, and leukemia) [8].

In this case, a lymph node biopsy was performed because it was difficult to exclude malignant lymphoma, although Kikuchi's disease was suspected in addition to pyogenic lymphadenitis based on the clinical course, blood tests, and imaging studies. Although Kikuchi disease was diagnosed by lymph node biopsy, the histological differential diagnosis of Kikuchi disease is mainly reactive lesions such as lymphadenitis associated with systemic lupus erythematosus, lymphadenitis caused by microorganisms such as herpes simplex, nonHodgkin's lymphoma, plasmacytic T-cell leukemia, Kawasaki disease, metastatic adenocarcinoma, or acute myeloid leukemia [9]. The characteristic pathology of Kikuchi's disease is the absence of neutrophilic infiltration in the necrotic areas [10]. The outcome is usually good, with spontaneous resolution of symptoms in most cases and recurrence in 3%–4% of cases. If no complications arise, the disease resolves within a few months. Symptomatic treatment is the mainstay of therapy, and there is no specific treatment. Corticosteroids are used only in severe cases or in cases of recurrence [10, 11].

In Kikuchi's disease, lymphadenopathy is most commonly located in the neck. Almost all patients with pathologically diagnosed Kikuchi disease had cervical lymphadenopathy [6, 7].

In order to search case reports of Kikuchi's disease with axillary lesions, we checked the academic article database PubMed and the Japanese article database Ichushi with the key words “Kikuchi's disease and/or axillary lymphadenopathy” and “histiocytic necrotizing lymphadenitis and/or axillary lymphadenopathy.” Mannu et al. reported a 19-year-old girl with no underlying disease who visited the hospital because of a mass in the right axilla that had been noted 2 months earlier [12]. There were no lesions in the breast. After an excisional biopsy of the axillary lesion, she was diagnosed with Kikuchi's disease. In the case of Verroiotou et al., a 56-year-old man with no underlying disease presented with left axillary lymphadenopathy [13]. Norris et al, reported a 63-year-old man with diabetes mellitus who was admitted to the hospital with fatigue, myalgia, a fever, and weight loss that persisted for 4 weeks [14]. Facial flushing and bilateral axillary lymphadenopathy were noted, as well as a decreased white blood cell count, elevated LDH, and elevated erythrocyte sedimentation. An axillary lymph node biopsy revealed a diagnosis of Kikuchi's disease.

## 4. Conclusion

To our knowledge, this is the only pediatric case of Kikuchi's disease presenting exclusively with axillary lymph node involvement. Why lymphadenopathy in Kikuchi's disease is often located in the neck is unclear, but it should be noted that lymphadenopathy outside the neck may also be present in Kikuchi's disease.

## Figures and Tables

**Table 1:** Laboratory data on admission.

Parameter	Recorded value	Standard value
White blood cell count (/*μ*L)	4,400	4,500–13,000
Neutrophils (%)	65.8	
Hemoglobin (g/dL)	15.4	12.0–14.5
Hematocrit (%)	45.8	44–50
Platelet count (×10^4^/*μ*l)	29.5	15–40
ESR (mm/h)	33	1–10
Total protein (g/dL)	8.1	6.5–8.1
Albumin (g/dL)	4.4	3.8–4.8
Aspartate aminotransferase (U/L)	33	13–30
Alanine aminotransferase (U/L)	26	9–35
*γ*-glutamyl transpeptidase (U/L)	22	0–50
Creatinine (mg/dL)	0.75	0.48–0.93
Urea nitrogen (mg/dL)	11	6–20
Na (mEq/L)	137	138–145
K (mEq/L)	4.3	3.4–4.7
Cl (mEq/L)	98	98–106
C-reactive protein (mg/dL)	1.19	<0.2
CH50 (U/mL)	>60	25–48
C3 (mg/dL)	149	86–160
C4 (mg/dL)	56	17–45
*β*2-microglobulin (mg/L)	2.9	0.9–2.0
Serum amyloid A (*μ*g/mL)	236.3	<8.0
IgG (mg/dL)	1374	770–1690
IgM (mg/dL)	103	67–291
IgA (mg/dL)	211	78–376
T-spot *tuberculosis*	Negative	
Antinuclear antibody	Negative	
CMV-IgM	Negative	
VCA-IgM	Negative	
Serum IL-2 receptor (U/mL)	847	145–519
CD4/CD8 ratio	0.64	0.4–2.3
